# A case of intercommunity lethal aggression by chimpanzees in an open and dry landscape, Issa Valley, western Tanzania

**DOI:** 10.1007/s10329-023-01085-6

**Published:** 2023-08-24

**Authors:** Rhianna C. Drummond-Clarke, Caroline Fryns, Fiona A. Stewart, Alex K. Piel

**Affiliations:** 1 Department of Human Origins, Max Planck Institute of Evolutionary Anthropology, Leipzig, Germany; 2 Insitut de Biologie, Université de Neuchâtel, Rue Emile-Argand 11, 2000 Neuchâtel, Switzerland; 3 Department of Anthropology, University College London, London, UK; 4 School of Biological and Environmental Sciences, Liverpool John Moores University, Liverpool, UK

**Keywords:** Xenophobia, Violence, Infanticide, Resource competition, Great ape, Savannah

## Abstract

Intercommunity (lethal) aggression is a familiar component of the behavioural repertoire of many forest-dwelling chimpanzee (*Pan troglodytes*) communities. However, until now, the absence of intercommunity attacks – including killings – in communities that live in open, mosaic environments has supported hypotheses of reduced resource competition in drier habitats, and informed referential models of early hominin social dynamics in a similar habitat. In June 2020, we observed the first instance of intercommunity lethal aggression, a male-committed infanticide, by the Issa chimpanzee community, which live in a savannah-mosaic habitat in the Issa Valley, western Tanzania. The carcass was recovered by researchers after it was abandoned by the attackers. Here, we give a detailed account of the events leading up to and including the infanticide, and contextualise our observations with what has been described for other chimpanzee communities. Notably, in contrast to the majority of reported intercommunity infanticides, the infant male victim was castrated (and not cannibalised), making this the youngest reported castration. This observation of intercommunity aggression disproves its hypothesised absence in savannah-dwelling chimpanzees, which by extension, has implications for early hominin evolution. We suggest that the near absence of observations of intercommunity aggression in savannah chimpanzee communities is most likely due to the lack of long-term study communities, and in some cases geographic isolation. We hypothesise that food-rich areas within a habitat with otherwise widely distributed food sources may select for intense intercommunity aggression despite the low population density characteristic of savannah communities. Anecdotes such as this add to the comparative database available on intercommunity killings in chimpanzee society, improving our ability to draw inferences about their evolutionary significance.

**Supplementary Information:**

The online version contains supplementary material available at 10.1007/s10329-023-01085-6.

## Introduction

Comparisons between chimpanzee and human behaviour are an important method to model the social dynamics of the human–chimpanzee last common ancestor (LCA) and early members of the human lineage (hominins) (e.g. Muller 2017; Wilson and Glowacki 2017; Wrangham and Benenson 2017). Notably, wild chimpanzee and human hunter–gatherer groups exhibit similar rates of intergroup lethal aggression (Wrangham et al. 2006), but we cannot know whether similarities were inherited from the LCA or evolved convergently (Wilson and Glowacki 2017). Chimpanzees that live in habitats analogous to those reconstructed for early hominins – mosaic environments that are more open and arid than evergreen forests (van Leeuwen et al. 2020) – are suggested to make particularly informative models for early hominin behaviour due to shared ecology (Moore 1992, 1996; Hunt and McGrew 2002; Hernandez-Aguilar et al. 2007; Pruetz and Bertolani 2009; Pruetz and Herzog 2017; Lindshield et al. 2021; Hunt et al. 2021; Drummond-Clarke et al. 2022). Below, we report the first observation of an intercommunity lethal encounter in a savannah community of chimpanzees and discuss it in the context of our current understanding of chimpanzee lethal aggression.

Chimpanzee (*Pan troglodytes*) social dynamics are characterised by intra- and intercommunity hostility. Notably, intra- and intercommunity infant killing (hereafter ‘infanticide') have been documented since early observations of wild chimpanzees began (e.g. Suzuki 1971; Bygott 1972; Goodall 1977), and adult males are widely known to cooperate to conduct territorial patrols and kill strangers from neighbouring communities (Goodall 1979; Boesch and Boesch-Achermann 2000; Watts and Mitani 2001; Wilson and Wrangham 2003; Watts et al. 2006; Muller and Mitani 2005). Currently, intercommunity lethal aggression has been observed, or inferred, at eight different communities and in three of four subspecies (or rather, all subspecies with long-term observations), with male-committed intercommunity infanticide being particularly prevalent (Wilson et al. 2014).

The evolutionary origins of male-committed intercommunity lethal aggression in chimpanzees remains a key question in palaeoanthropology, with many hypotheses existing that are centred around adaptive strategies that increase male fitness, directly and indirectly, by improving access to resources – primarily food and females (e.g. Wrangham 1979; Wrangham and Smuts 1980; Wilson et al. 2014). The ‘exploitation hypothesis’ suggests that perpetrators increase fitness directly by gaining nutritional benefits from consuming the victim (Hrdy 1979); however, inconsistency in consumption rates of victims make this an unlikely motive for intercommunity killings (Kirchhoff et al. 2018). The ‘sexual selection hypothesis’ (Hrdy 1979; Nishida et al. 1979; Arcadi and Wrangham 1999), where the killing of an unrelated infant shortens postpartum subfecundity in the victim’s mother, would directly increase reproductive opportunity of a male attacker. In the case of intercommunity infanticides, the female could also be coerced to leave her own community and join the attacking group to improve the safety of her next infant (the ‘female coercion hypothesis’; Arcadi and Wrangham 1999). However, uncertainty about mating rates between male attackers and mothers of infanticide victims from other communities makes such hypotheses of direct sexual selection hard to test (e.g. Boesch et al. 2008). Alternatively, the ‘resource competition’ or ‘range expansion hypothesis’ suggests that intercommunity aggression deters extra-community members from border areas, leading to range expansion of the attacking group (Williams et al. 2004). This is hypothesised to increase male fitness indirectly through expanding the area where females in their own community can forage safely, thereby increasing resident female fitness and the potential for their own (male) reproductive success (Pusey 2001; Watts et al. 2002; Williams et al. 2004), and is supported by observations of decreased inter-birth intervals of resident females with range expansion at Gombe (Williams et al. 2004; Pusey et al. 2005). A larger home range is also hypothesised to have the secondary effect of increased immigration of new females (Watts and Mitani 2000; Watts et al. 2002; Williams et al. 2004; Sherrow and Amsler 2007).

When the attacker(s) and victim are male (as is the majority in reported intercommunity attacks; Wilson et al. 2014), rival reduction may also explain killings (e.g. Arcadi and Wrangham 1999; Newton-Fisher 1999; Kutsukake and Matsusaka 2002; Watts et al. 2002). A link to male rival reduction may also be supported by a high frequency of castration of adolescent/adult male victims in intercommunity attacks, if we can assume this indicates knowledge of the role of the testes in reproduction (e.g. Wilson et al. 2015).

In parallel to the above hypotheses, the ‘imbalance of power hypothesis’ predicts the likelihood of a violent encounter increases when one party greatly outnumbers the other (or victim’s) party (Manson and Wrangham 1991; Wrangham 1999), and has support from observations of intercommunity violence across multiple sites (Sherrow and Amsler (2007) and references there-in).

Despite high variability in mortality rates caused by intercommunity killings across forest-dwelling communities (see Table 3 in Boesch et al. 2008), until now, intercommunity killings have never been documented in a savannah-dwelling community. Of the few chimpanzee studies conducted in arid, open habitats, most have been short term and/or not fully habituated (e.g. Mt Assirik and Semliki; Hunt and McGrew 2002; Hunt 2020), or the study community is suggested to be geographically isolated from neighbouring communities (e.g. Fongoli; Pruetz et al. 2017), posing obvious limitations on the observation or occurrence of intercommunity interactions of any kind. However, it is notable that savannah chimpanzee communities are characterised by larger home ranges with more sparsely distributed food sources than their forest dwelling counterparts (Moore 1992; Lindshield et al. 2021). Combined with population density being a significant predictor of lethal aggression rates (increased density = increased aggression; Wilson et al. 2014), this lack of observations in savannah-dwelling communities offers support for early hypotheses related to the costs to social organisation in savannah chimpanzees (sensu Moore 1992, 1996), including predictions that lower population density leads to less territoriality in savannah-mosaic communities (e.g. Samson and Hunt 2014). If so, we would expect overlapping ranges, combined with far less, or an absence of, intercommunity aggression.

Here, we report the first observed intercommunity killing between chimpanzees in a savannah-mosaic habitat in the Issa Valley, western Tanzania, and along with it the youngest ever recorded castration in chimpanzee intercommunity aggression. We contextualise the timeline and observations with previously described intercommunity killings and discuss the implications for chimpanzee territoriality and human evolution.

## Methods

### Study site and subjects

The Issa study site is situated in western Tanzania in the Greater Mahale Ecosystem. It constitutes ~85 km^2^ comprised of five major valleys separated by steep mountains and flat plateaus ranging from 1050 to 1750 m in elevation (Giuliano et al. 2022). The region is classed as a savannah-mosaic landscape, dominated by miombo woodlands (*Brachystegia* and *Julbernardia* spp.) interspersed with rocky outcrops, riparian forest and patches of seasonally inundated grassland (Piel et al. 2017; Drummond-Clarke et al. 2022). Issa is one of the driest and most seasonal habitats for chimpanzees (van Leeuwen et al. 2020; Lindshield et al. 2021). The wet season (April–October) includes nearly all annual rainfall (rainfall averages 1205 mm/year, range 717–1747 ml from 2009 to 2021), with the dry season (monthly rainfall < 100 mm) lasting over 6 months from May to October, marked by annual grass fires predominantly set by humans that burn more than 75% of the landscape (Piel and Stewart unpublished data).

**Fig. 1:**
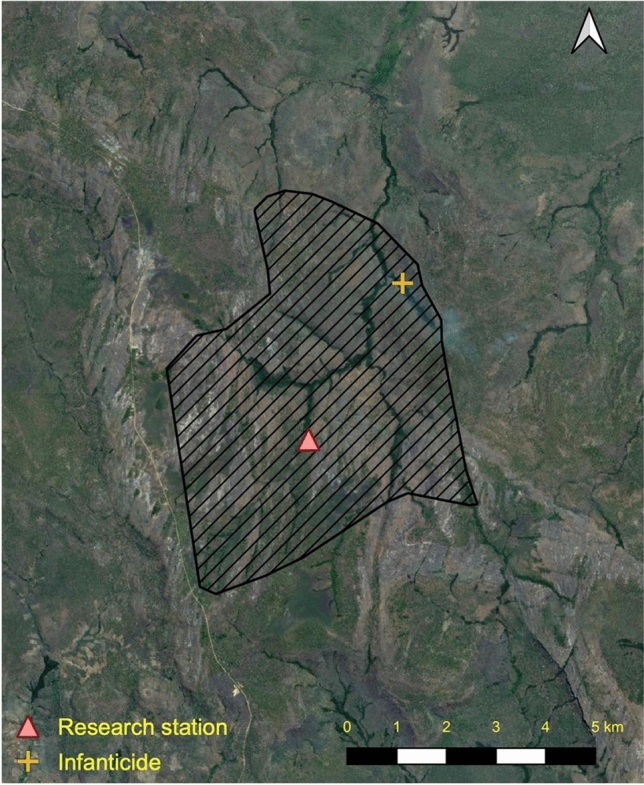
Location of the intercommunity fatal encounter (yellow cross) in relation to the research station (pink triangle) and the habituated Issa community’s known home range (black lines). Valleys (dark green strips) are lined with riparian forest, the plateaus are dominated by miombo woodland. The infanticide took place at the intersect of Issa Valley (long valley in north south orientation) and Mgumu Valley (shorter connecting valley). The attacking party remained within 800 m of the attack site for at least 3 h after the attack took place, resuming normal foraging and grooming behaviour

There has been a continuous research presence at Issa since 2008, with the Issa community considered fully habituated (nest-to-nest follows) as of mid-2018. At the time of the current observation, the community consisted of 27 individuals (seven adult males, eight adult females, three adolescent males, two adolescent females, four juveniles, three infants). For this study, infants were classified as unweaned individuals estimated between 0 and 4 years old (yo), juveniles 5–9 yo, adolescents 10–15 yo and adults 16 yo and over or after first pregnancy for females (we estimated all ages). The current home range is estimated at a minimum of 39 km^2^ (Piel and Stewart, unpublished data; Fig. 1).

### Data collection

The first author and a field assistant (hereafter referred to as ‘the researchers’) conducted a focal follow (Altmann 1974) on juvenile male MO, beginning at 07:05 h on the morning of 4 June 2020. At the time, research teams collected focal and group scan data for long-term research into chimpanzee’s behaviour, diet and ranging patterns. They also collected ad libitum video recording (using Sony FDR-AX53 camcorder) whenever possible of unusual behaviours, in this case the intercommunity encounter, allowing for an accurate timeline and detailed account. The researchers followed the chimpanzee party until 12:30 h. The carcass was retrieved at 14:00 h after being abandoned by the party, and was returned to the station where it was photographed and buried for later retrieval of the skeleton. The age of the infant victim was estimated from its size and dependency on its mother. An ethogram of observed behaviours is provided in Table S1.

## Description of intercommunity lethal encounter, 4 June 2020

06:20h: The researchers arrived at the nest site of eight males (four adults, three adolescents and one juvenile; Table 1), in the periphery of the known Issa community home range (Fig. 1), with the entire party still in their nests.

**Table 1:** Party composition and role in the attack

	ID	Sex	Age	Rank	Role
**Victims** Likely from northern community^a^	UID 1	Female	Adult	–	Mother of infant^b^. Tried to regain infant, suffered minor injuries, left after apparent death of infant
	UID 2	Male	Infant (approx. 2–3 yo)	–	The victim of the killing
**Attacking Party** Issa-habituated community	IM	Male	Adult	Alpha	Participated in killing
	WA	Male	Adult	High	Main participant in killing
	KIT	Male	Adult	High	Participated in killing, carried body after death
	SAM	Male	Adult	Middle	Participated in killing
	SA	Male	Adolescent	–	Main participant in killing
	WI	Male	Adolescent	–	Observed events close by, interacted with carcass (grooming, smelling)
	DH	Male	Adolescent	–	Observed events from a distance, interacted with carcass (grooming, smelling)
	MO	Male	Juvenile	–	Observed events from a distance, interacted with carcass (grooming, smelling)
	BO	Male	Adult	High	Arrived after infanticide finished and group moved away from scene. Interacted with carcass (grooming, smelling)
	KU	Male	Juvenile	–	Arrived after infanticide finished and group moved away from scene. Interacted with carcass (grooming, smelling)

^a^Caught on northern camera trap in May before event (Fig. S1)

^b^Assumed mother since chimpanzees stay with their mothers consistently until weaning, around 5 years of age (Lonsdorf et al. 2020), and the likely pair were caught on camera trap, together, in May before event (Fig. S1)

**07:10 h**: The party, led by IM, began pant-hooting and ran into a riparian forest strip.

**07:20 h:** The entire party fed on fruit in a *Cordia* sp. tree.

**07:34 h:** The party changed feeding trees to a *Ficus* sp.

**08:00 h**: The party left the tree and began walking north quickly and quietly. Adolescent male SA was the last to leave the tree, and walked separately from the party, staying behind in the forest, but moving in the same direction. After around 5 min, multiple individuals vocalised, and were answered by SA, who was about 30 m behind the main party. At this point, researchers were behind with SA (since they could not bypass him on the thin forest path), and could not see the main party.

**08:09 h:** Following the vocalisations, the researchers left SA and found the other males in the woodland, adjacent to the forest strip, where the males had encountered an unidentified adult female and infant. Five males (IM, SAM, WI, DH, MO) were in a tree and two (KIT and WA) were beneath them in the grass, with all individuals vocalising (pant–hoots, barks and screams). The (arboreal) males abruptly descended, and the researchers could hear the screams of an infant chimpanzee. The researchers could see constant displays. After a few minutes, SA pant–hooted from the forest strip, and multiple adult males in the party replied, then IM, WA and KIT ran in the direction of SA’s pant–hoot, returned quickly with SA. Vocalisations and displaying continued for 15 min, with SAM, WI and DH watching the others from several metres above in a nearby tree.

**08:27 h:** WA climbed up a nearby tree, and the researchers saw he was holding an infant chimpanzee (estimated to be between 2 and 3 yo) against his chest, with the infant’s hand in WA’s mouth (Supplementary video 1). DH and WI followed WA mid-way up the tree.

**08:28 h:** WA was joined by SAM, SA and IM, with DH and an unknown female (the infant’s mother) just below them in the tree. SA, IM and KIT displayed, SA and WA bit the infant, and SA bit the infant’s penis and scrotum off (Supplementary video 2). The infant was screaming throughout. The infant’s mother attempted to retrieve the infant repeatedly, reaching for its legs before and after the castration, but she was always deterred by KIT who poked the female while WA and SA push her back, and displays by IM. At this point the researchers noticed the female had a swollen, bruised eye, as well as a fresh cut on her buttocks. WA climbed higher in the tree, continuing to hold the infant by the hand in his mouth, while the female barked as the males screamed and hooted. Adolescent WI also climbed up the tree and briefly touched the infant. As WA climbed the tree further, the infant grabbed hold of a branch. WA pulled the infant forcing him to let go of the branch.

**08:29 h:** SA climbed and sat next to WA, as they renewed attacks on the infant, SA focused on the limbs, biting, pulling, and bending, while WA bit the infant’s abdomen. IM then displaced SA, but did not engage with the (still conscious, but severely wounded) infant. KIT approached and grabbed one of the infant’s feet, while WA held the infant and bit his pelvic area and then the throat. Throughout this period, there were screams, hoots and barks, and the female was not visible but could be heard barking from the ground (Supplementary video 2).

**08:30 h:** SAM joined the attacking group, bringing the attacking party to five (SAM, SA, WA, IM, KIT). SA and WA bit the infant’s head, while WI bit the infant’s foot and SAM the back. No chewing was observed. IM began hooting and displayed towards WI, who retreated. At this point, weak screams from the infant and barks, likely coming from the female below, could still be heard. IM displaced SAM and targeted the legs/genital area/stomach. WA, SA, IM and KIT continued to bite and tug the infant until his limbs were limp, at this point researchers assumed the infant was dead (Supplementary video 2).

**08:31 h:** The female (who had climbed up an adjacent tree and back into view) climbed down (passing MO who was sitting just below), and moved out of site on the ground, hooting and barking as she left. After this point, she was not seen again (Supplementary video 3). During this time, KIT, IM, SAM, SA and WA were clustered in the tree, SA and WA still holding on to the body. SA bit and pulled on the limbs, while WA appeared to bite the genital area. KIT attempted to take the carcass from WA but failed, while SA and WA continued to bite it. MO sat separately (approx. 8 m) from the older males, but watched the males and carcass.

**08:34 h:** WA still held the body, but SA, SAM and KIT continued to pull limbs and appeared to bite it, and IM bit the genital area, but did not try to take the carcass from WA or stop others interacting with it (Supplementary video 3).

**08:37 h:** KIT took the body from WA, and began to groom the thigh, as WA peered at him (Supplementary video 3).

**08:38 h:** WA took back hold of the torso and KIT, IM, SA and SA each held part of the carcass. SA bit the right hand, IM bit the genital area, and WA, SA, SAM and KIT continued smelling and biting the carcass (Supplementary video 3).

**08:41 h:** KIT pried the carcass from WA and began climbing the tree. SAM, WA, IM and SA followed. SAM and WA tried to interact with the carcass, and WA briefly succeeded and appeared to bite it, then left and climbed further up the tree (Supplementary video 3).

**08:42 h:** IM started a pant-hoot, and others joined in to chorus, during which SAM and KIT moved away from IM. After a few seconds the pant–hooting stopped, and KIT moved further along the branch with the infant’s body, followed by SAM and WA. SAM looked at and touched the castration area, and WA the upper body. IM and SA also approached and held the carcass. IM, SA and WA continued to bite and inspect the hands, upper body and genital area while KIT held on to the carcass (Supplementary video 3).

**08:44 h:** KIT still had hold of the legs, and SA, IM and WA were still biting the upper body and pelvic area. WI then approached from below, appeared to bite down once on the infant and then retreated. DH and MO sat below the other males in the same tree, but were not showing interest in the scene (Supplementary video 4).

**08:45 h:** IM moved away and the carcass dropped to the ground from their location around 8 m high in the tree). The first to follow and retrieve the carcass was KIT, with WA, IM and SAM following. DH and MO remained in the tree, watching the others on the ground (Supplementary video 4).

**08:46 h:** KIT climbed back up the tree with the carcass, followed by SAM, WA, DH and SA. KIT stopped and sat, and MO approached KIT and peered at the carcass, then moved away. After 45 s, KIT resumed climbing with the carcass followed by WA, IM, SAM, DH and SA. WA and IM moved close to KIT and the carcass. WI followed from approx. 4 m (Supplementary video 5).

**Fig. 2:**
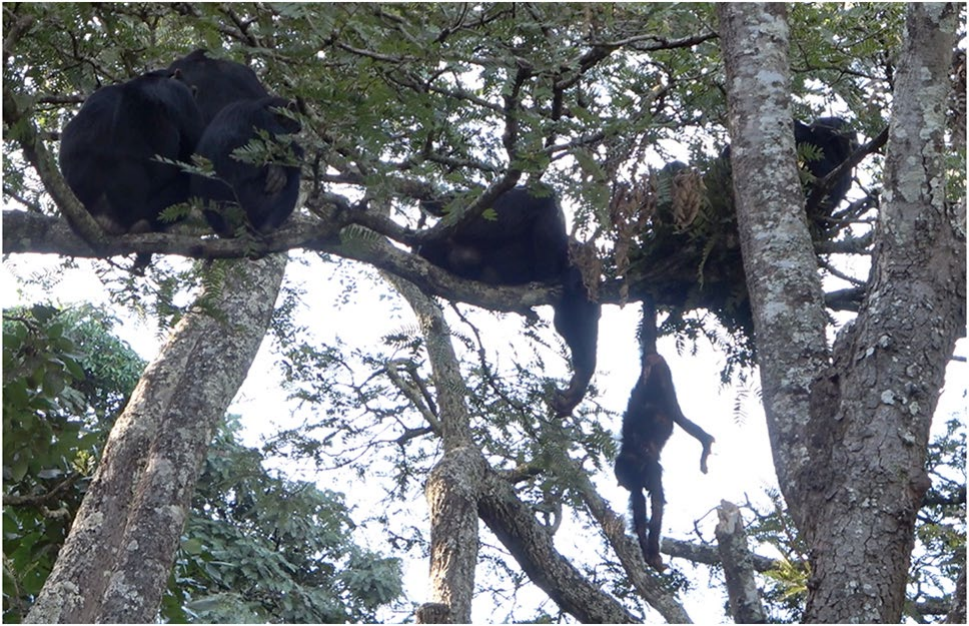
Adult, adolescent and juvenile males from the attacking party with the infant’s carcass shortly after the attack. KIT is in a nest made minutes before by him, holding the infant’s carcass (dangling below). WA is showing interest in the carcass but does not take it back from KIT after this point. Screenshot taken from Supplementary video 7 (see Supplementary videos 6 and 7 for nest-making sequence)

**09:00 h:** KIT moved a metre away from the others with the carcass and made and sat in a nest, letting the carcass dangle by the foot (Fig. 2; Supplementary videos 6, 7). WA moved closer to KIT, and dangled his arm next to the carcass but did not touch it (Fig. 2).

**09:03 h:** KIT left his nest and climbed down the tree, stopping at the same level as MO on another branch. WI moved to KIT and grabbed the pelvis and brought his face to the carcass to bite or smell the area. After a few seconds KIT moved the carcass away from WI, but WI moved closer in and put his lips to the genital area, but did not bite, then moved away to just below KIT and was joined by MO (Supplementary video 8).

**09:04 h:** KIT swung down, leaving the carcass in the tree above. KIT then reached for a limb of the carcass, and pulled it down, then climbed to the ground dragging the carcass. WI and MO followed. All other chimpanzees were already on the ground (Supplementary video 8).

**09:11 h:** The party was travelling cardinal direction through the riverine forest strip for about 100 m. KIT dragged the carcass along the ground behind him (Supplementary video 9).

**09:18 h**: The party stopped, and after a quiet pause, began displaying, leaving the carcass and crossing over a stream. When they returned to the original spot where the carcass remained, still displaying, they were accompanied by two other community members, BO (adult male) and KU (juvenile male), who had not previously been seen that day. The party continued displaying all together for a few minutes– pant–hooting, running, buttress root drumming – then went calm. BO briefly inspected the carcass, looking and smelling, then BO groomed with the other adults. KU, MO, DH and WI examined the carcass on the forest floor.

**09:30 h:** The party travelled south, leaving the carcass behind. They passed the location where the infanticide had taken place but did not appear to react. At this point, the researchers briefly inspected the infanticide location, which was easily distinguishable by the flattened grass, and saw a small pool of blood on the grass.

The researchers stayed with the party until 12:30 h while they regularly groomed, rested and fed on fruit as they continued to travel south along a valley towards the centre of their known home range.

**Fig. 3:**
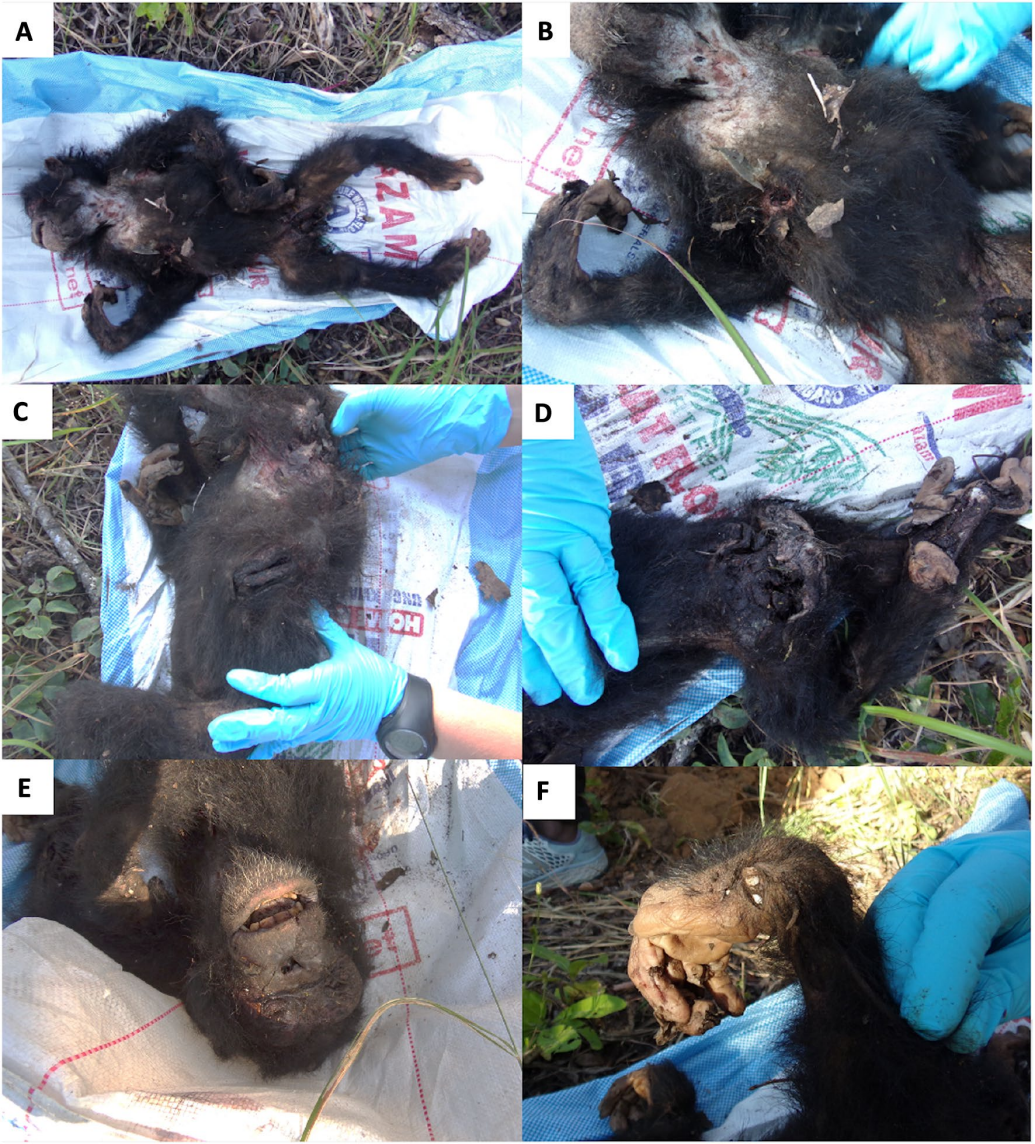
Post-mortem photos of the injuries incurred by the attacking males on the infant victim during the intercommunity lethal encounter. Injuries incurred include: Broken right femur and tibia (**A**), removed genitalia (**A, B**), punctured and lacerated throat and chest (**A, B**), a large gash in the left ribs (**C**), ripped skin from the left foot and deep wounds on the right hip (**D**), a deep laceration to the face below the left eye (**E**), deep wound (to the bone) on the right hand, and ripped skin on the fingers (**F**)

Although we cannot completely rule out consumption of any kind (i.e. of missing genitals), during all above-described events we did not observe any targeted and seemingly intentional feeding for nutritional purposes, and apart from severe wounds (gashes to the head, neck and torso, lacerations to the hands, legs, and feet, and the missing genitalia, see Fig. 3), the infant’s body was otherwise intact upon retrieval by the researchers that afternoon.

## Discussion

Our observation of intercommunity lethal aggression in the Issa Valley is the first to be observed in a savannah-mosaic community of chimpanzees. Although more data are needed on overall intercommunity encounter rates in savannah-mosaic chimpanzees, this observation falsifies the hypothesis that intercommunity killing does not occur in an open and dry, or low population density context (Samson and Hunt 2014; Wilson et al. 2014).

### Comparison with previous reports of lethal intercommunity encounters

Here, a party of eight males participated in an infanticide involving the castration of an infant male (estimated 2–3 yo) from a neighbouring community following an attack on the infant and his likely mother, in a peripheral area of the Issa community’s known home range (Fig. 1). To our knowledge, they did not cannibalise the dead infant, although they maintained control of the carcass for around 1 h after the victim’s death, and remained in the area to forage, rest and groom. The mother eventually left the attacking group and infant’s carcass without any serious injuries. This case of lethal intercommunity aggression bears resemblance to multiple cases already described in forest communities in several ways.

First, as with most intercommunity aggressions, the attack took place in the periphery of the attacking community’s known home range, and the attacking party (eight males, of which seven were adult or adolescent) greatly outnumbered the victims (two individuals, one adult female and her infant), resulting in a ratio very similar to the 8:1 median of adult and adolescent individuals in each party calculated from other attacks (Wilson et al. 2014; Wilson and Glowacki 2017). Additionally, the mixed age range of the all-male group of attackers was consistent with the party composition of cases reported at Taï (Boesch et al. 2008), Mahale (Kutsukake and Matsusaka 2002), and Ngogo (Watts and Mitani 2000). Second, the victims were a lone female and her unweaned dependent, the infant being the focus of the attack as is the case in just over half of observed intercommunity killings (Wilson et al. 2014; Wilson and Glowacki 2017) and further, the infant was male; males account for over 78% of reported intercommunity aggression fatalities (Wilson et al. 2014). Despite these similarities between the current and published accounts, differences exist.

Foremost, this case differed from many intercommunity lethal attacks on sexually immature individuals in that the victim was castrated (Fig. 3A, B). Although commonly described for attacks on sexually mature males (e.g. Wilson et al. 2015), there is only one other reported possible castration of an infant during an intercommunity attack, observed in the Ngogo community (Uganda; Watts and Mitani 2000). However, the sex of the infant was unknown and it was completely cannibalised by the attackers, making castration uncertain (Watts and Mitani 2000). As such, our observation represents the youngest confirmed castration (without cannibalisation) by chimpanzees. Second, although the victim suffered substantial bite wounds and castration, the attackers were not observed to cannibalise the body (also confirmed by the carcass being complete, except the missing genitals, on retrieval). While cannibalism is rare in intercommunity attacks on adult victims, it is common on infant victims (including older infants between 1.5 and 3 yo), especially when the attackers remain where the encounter took place (Arcadi and Wrangham 1999; Kirchoff et al. 2018). Finally, in most cases of chimpanzee intercommunity aggression, the attacking party flee after the victim is wounded or dead (Watts et al. 2002). In the current case, however, the attacking party remained within 800 m of the attack for over 3 h, one of the males even making a day nest to sit in with the carcass at the site of the attack (Fig. 2). We cannot be sure why in some cases attackers retreat and in others they remain, but given the potential for intercommunity aggression, it is likely that no males from the neighbouring community were nearby, so there was minimal threat of (counter-) attack (e.g., Watts et al. 2002). It is also possible that researcher presence deterred non-habituated chimpanzees from approaching (e.g. Tutin and Fernandez 1991; Bertolani and Boesch 2008).

### Adaptive strategy

More observations of intercommunity encounters are clearly needed to test hypotheses about selective pressures for intercommunity lethal aggression. While this observation falsifies the hypothesis that intercommunity killing by chimpanzees occurs only in high-density forest settings, without more observations we cannot know whether rates of intercommunity aggression are similar at Issa compared with other chimpanzee communities. However, we can compare this observation with other published cases to assess whether there could be support for previously proposed hypotheses. This observation does not support the exploitation hypothesis (Hrdy 1979), as the victim was not consumed after death. It does however lend support to several different hypotheses.

First, it supports the ‘range expansion hypothesis’ as it took place (i) on the periphery of a (known) home range where researchers have observed members of the neighbouring chimpanzee community (unpublished data) and (ii) in a forest strip with multiple known feeding trees (*Cordia* sp. and *Ficus* sp*.*), during a period of seasonal food scarcity (Wilson and Glowacki 2017; Giuliano et al. 2022). Second, that the attacking party greatly outnumbered the victims supports the ‘imbalance of power hypothesis’, where the larger the attacking party, the lower the cost of attack (Manson and Wrangham 1991; Sherrow and Amsler 2007). Third, that the victim was male, and castrated early in the attack lends support to the ‘male–male competition’ (or rival reduction) hypothesis (Arcadi and Wrangham 1999; Newton-Fisher 1999; Kutsukake and Matsusaka 2002; Watts et al. 2002). Further, castrating the male victim, despite his sexual immaturity, supports the ‘resource defence’ and ‘rival reduction’ hypothesis in that infanticide is functionally equivalent to the killing of adult males (Wilson et al. 2004), where castration is common during intercommunity aggression (e.g. Boesch et al. 2007; Wilson et al. 2015). Although the killing of an infant by males from another community may lend support to the ‘sexual selection’ or ‘female coercion hypothesis’, it is difficult to comment on this without future observation of the female’s ranging and mating patterns. It should however be noted that the female has not been seen with the habituated (attacking) community since the infanticide took place (3 years at the time of writing), providing no evidence that she transferred to the attacking community as a result of the infanticide/coercion.

### Implications for chimpanzee behavioural ecology in a savannah-mosaic landscape

There is substantial variation in intercommunity encounter and lethal aggression rates across chimpanzees (Boesch et al. 2008; Wilson et al. 2014). Issa’s single observation of intercommunity lethal aggression after 4 years of behavioural follows is not a low rate compared to other long-term chimpanzee study sites, despite a low population density (Issa rate = 0.25 observed killings/year versus a reported median of 0.24 (min = 0, max = 1.83), calculated from Table 3 in Boesch et al. 2008). This holds even when considering only eastern chimpanzee communities, which are considered more aggressive than the western subspecies based on observations of forest-dwelling communities (Boesch et al. 2008; Wilson et al. 2014). Savannah-mosaic chimpanzees are characterised by ecological temporal and spatial heterogeneity with key foods overall more sporadic, but also more condensed (principally in riparian forest strips) during food scarce times of the year (van Leeuwen et al. 2020; Lindshield et al. 2021). Specifically at Issa during the food scarce period that this intercommunity encounter took place, chimpanzees were observed feeding principally on *Cordia* and *Ficus* spp. fruit, both of which occur in forest and have high nutritional value (Piel et al. 2017; Giuliano et al. 2022). Abundant supplies of food in border areas increase the likelihood of intercommunity encounters in forest settings by attracting chimpanzees from both sides of the boundary (Wilson et al. 2012). We therefore hypothesise that increased food sporadicity characteristic of dry habitats could further increase the likelihood of intercommunity encounters (and competition) at high-quality, food-rich patches (e.g. forest strips), counter-balancing negative effects of low population density on encounter rates during times of food scarcity.

Chimpanzees from the Fongoli community (Senegal) are the only other fully habituated savannah-dwelling chimpanzees with long-term observations. Despite 15 years of study at Fongoli, there have been no descriptions of intercommunity encounters. Importantly, Issa and Fongoli differ in taxonomy: Issa Valley hosts the eastern subspecies (*P. t. schweinfurthii*) and Fongoli the western subspecies (*P. t. verus*). Although observations of intercommunity interactions in western chimpanzees are largely limited to Taï Forest (due to Taï being the only western site with long-term observations and neighbouring communities), eastern chimpanzees have been described as less cohesive than their western counterparts, and this has been linked to higher rates of intercommunity aggression reported in the eastern subspecies (i.e. smaller parties are more vulnerable to attack, in relation to the imbalance of power hypothesis; Wrangham 1999; Boesch et al. 2008; Wilson et al. 2014). However, in these open habitats individuals from both communities exhibit comparatively high gregariousness in grouping behaviour (Pruetz and Bertolani 2009; Giuliano et al. 2022). This suggests that differences in intercommunity aggression rates between eastern and western communities cannot be linked to cohesiveness alone, forcing us to reconsider the roles of taxonomy and habitat on chimpanzee behaviour. It is notable that environmental differences exist between Issa and Fongoli that may affect intercommunity encounter rates. In particular, the Fongoli community is suggested to be geographically isolated from neighbouring communities by anthropogenic (a paved road and settlement) and natural (Gambia River) features (Pruetz et al. 2017). This not only imposes obvious limitations on the occurrence of intercommunity encounters (and thus aggression) at Fongoli, but is markedly in contrast to the Issa community, for which camera trap footage and field observations (including the encounter reported here) reveal at least one neighbouring community, and likely two (unpublished data).

More data are needed on seasonal ranging patterns and intercommunity encounter rates at Issa (and other savannah-mosaic habitat chimpanzee sites) to further test the role of food rich patches on counterbalancing negative effects of a low population density on encounter rates. Our observations could however lend support to intense competition in food rich patches in a savannah habitat, in that it took place in a forest strip with abundant fruiting trees during a time of food scarcity at Issa (Piel et al. 2017; Giuliano et al. 2022). More observations of intercommunity encounters will be critical in understanding the differences in intercommunity group dynamics between chimpanzees across their ecological range.

### Supplementary Information

Below is the link to the electronic supplementary material.

Supplementary file1 (DOCX 16 KB)

Supplementary file2 (PDF 510 KB)

## Data Availability

All data used in this study are included in the published article and its supplementary information files.
