# Supplementary material for: A case of intercommunity lethal aggression by chimpanzees in an open and dry landscape, Issa Valley, western Tanzania
Source: Primates. 2023 Aug 24;64(6):599–608. doi: 10.1007/s10329-023-01085-6 (PMC10651548; doi:10.1007/s10329-023-01085-6)
Supplement: Supplementary file 1 — Supplementary file1 (DOCX 16 KB) [file 10329_2023_1085_MOESM1_ESM.docx]

**Supplementary videos for:**

**A case of intercommunity lethal aggression by chimpanzees in an open and dry landscape, Issa Valley, western Tanzania**

Rhianna C. Drummond-Clarke*^1^, Caroline Fryns^2^, Fiona A. Stewart^1,3,4^, Alex K. Piel*^1,3^

**Affiliations**

^1^ Department of Human Origins, Max Planck Institute of Evolutionary Anthropology, Leipzig, Germany

^2^ Insitut de Biologie, Université de Neuchâtel, Rue Emile-Argand 11, 2000 Neuchâtel, Switzerland

^3^ Department of Anthropology, University College London, London, UK

^4^ School of Biological and Environmental Sciences, Liverpool John Moores University, Liverpool, UK

*Corresponding authors’ emails: rhianna_drummond_clarke@eva.mpg.de (RCDC), [a.piel@ucl.ac.uk](mailto:a.piel@ucl.ac.uk) (AKP)

Please follow this link for the supplementary videos 1-9:

<https://www.dropbox.com/sh/gy1vegqe7tiaqar/AAAf_F2ofq1YNPW-_EaZRIc8a?dl=0>
