# Supplementary material for: A case of intercommunity lethal aggression by chimpanzees in an open and dry landscape, Issa Valley, western Tanzania
Source: Primates. 2023 Aug 24;64(6):599–608. doi: 10.1007/s10329-023-01085-6 (PMC10651548; doi:10.1007/s10329-023-01085-6)
Supplement: Supplementary file 2 — Supplementary file2 (PDF 510 KB) [file 10329_2023_1085_MOESM2_ESM.pdf]

Supplementary Materials

A case of intercommunity lethal aggression by chimpanzees in an open and dry landscape, Issa Valley, western Tanzania

Rhianna C. Drummond-Clarke*[1], Caroline Fryns[2], Fiona A. Stewart[1,3,4], Alex K. Piel*[1,3]

Affiliations

[1] Department of Human Origins, Max Planck Institute of Evolutionary Anthropology, Leipzig, Germany

[2] Insitut de Biologie, Université de Neuchâtel, Rue Emile-Argand 11, 2000 Neuchâtel, Switzerland

[3] Department of Anthropology, University College London, London, UK

[4] School of Biological and Environmental Sciences, Liverpool John Moores University, Liverpool, UK

*Corresponding authors' emails: rhianna_drummond_clarke@eva.mpg.de (RCDC), a.piel@ucl.ac.uk (AKP)

Contents:

Fig S1
Table S1
Supplementary videos 1-9
Supplementary reference 1

Fig. S1 Image of assumed mother and dependant infant carried dorsally, the victims of the intercommunity encounter, in May 2020 (the month before the attack), from a camera trap in the northern area of the Issa Valley, and north of the habituated communities known home range.

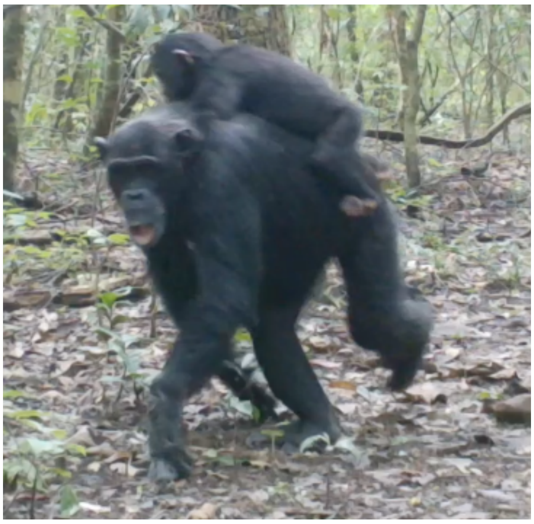

Table S1 Ethogram of behaviours observed during the intercommunity lethal encounter.

Behavior	Description
**Display**	Intimidating behaviour, hair is pilo-erect and may include swaying, vocalisations (pant-hooting, screaming), object shaking/throwing. Can be targeted at an individual or non-targeted.
**Attack**	Physical contact with aggressive motive, can include some or all of the following: hitting, slapping, biting, scratching, grabbing, jumping (on the attacked).
**Interact**	Close or physical contact with the subject, not aggressive, more inquisitive. E.g., Smell, touch (poke, hold, grab), groom, look closely.
**Vocalisation**[a]	Pant-hoot - A structurally complex vocalisation consisting of introduction, build-up, climax and let-down. Practiced mainly by adult males and during display and social excitement.<br>Hoot – low frequency 'hoo' calls, often used by adults in competitive feeding or begging contexts, as a distress signal.<br>Scream – loud, high-pitched vocalisation, used during agnostic encounters by victims and lower ranking attackers. Agnostic screams used to recruit aid.<br>Bark – sharp, loud and low-pitched calls. Often used by females more than males, in situations of social excitement and agnostic encounters. Function is unclear.
**Cannibalise**	Consummation of flesh and bodily parts.
**Castrate**	Targeted removal of the genital organs.

[a] Definitions from Table 16.1 in Lonsdorf et al. (2010)

Auxiliary supplementary materials:

Supplementary videos 1-9

Supplementary references:

Lonsdorf EV, Ross SR, Matsuzawa T (2010) The mind of the chimpanzee: ecological and experimental perspectives. The Univ of Chicago Press, Chicago and London.
